# Improvements in access to malaria treatment in Tanzania following community, retail sector and health facility interventions -- a user perspective

**DOI:** 10.1186/1475-2875-9-163

**Published:** 2010-06-15

**Authors:** Sandra Alba, Angel Dillip, Manuel W Hetzel, Iddy Mayumana, Christopher Mshana, Ahmed Makemba, Mathew Alexander, Brigit Obrist, Alexander Schulze, Flora Kessy, Hassan Mshinda, Christian Lengeler

**Affiliations:** 1 Dept. Epidemiology and Public Health, Swiss Tropical and Public Health Institute, Basel, Switzerland; 2 University of Basel, Basel, Switzerland; 3 Ifakara Health Institute, Ifakara, Tanzania; 4 Papua New Guinea Institute of Medical Research, Goroka, Papua New Guinea; 5 Novartis Foundation for Sustainable Development, Basel, Switzerland; 6 Tanzanian Commission for Science and Technology, Dar es Salaam, Tanzania

## Abstract

**Background:**

The ACCESS programme aims at understanding and improving access to prompt and effective malaria treatment. Between 2004 and 2008 the programme implemented a social marketing campaign for improved treatment-seeking. To improve access to treatment in the private retail sector a new class of outlets known as accredited drug dispensing outlets (ADDO) was created in Tanzania in 2006. Tanzania changed its first-line treatment for malaria from sulphadoxine-pyrimethamine (SP) to artemether-lumefantrine (ALu) in 2007 and subsidized ALu was made available in both health facilities and ADDOs. The effect of these interventions on understanding and treatment of malaria was studied in rural Tanzania. The data also enabled an investigation of the determinants of access to treatment.

**Methods:**

Three treatment-seeking surveys were conducted in 2004, 2006 and 2008 in the rural areas of the Ifakara demographic surveillance system (DSS) and in Ifakara town. Each survey included approximately 150 people who had suffered a fever case in the previous 14 days.

**Results:**

Treatment-seeking and awareness of malaria was already high at baseline, but various improvements were seen between 2004 and 2008, namely: better understanding causes of malaria (from 62% to 84%); an increase in health facility attendance as first treatment option for patients older than five years (27% to 52%); higher treatment coverage with anti-malarials (86% to 96%) and more timely use of anti-malarials (80% to 93-97% treatments taken within 24 hrs). Unfortunately, the change of treatment policy led to a low availability of ALu in the private sector and, therefore, to a drop in the proportion of patients taking a recommended malaria treatment (85% to 53%). The availability of outlets (health facilities or drug shops) is the most important determinant of whether patients receive prompt and effective treatment, whereas affordability and accessibility contribute to a lesser extent.

**Conclusions:**

An integrated approach aimed at improving understanding and treatment of malaria has led to tangible improvements in terms of people's actions for the treatment of malaria. However, progress was hindered by the low availability of the first-line treatment after the switch to ACT.

## Background

The cornerstone of the World Health Organization's malaria control strategy is prompt and effective treatment for all episodes of malaria [1,2]. Largely thanks to international support through the Global Fund to Fight AIDS, Tuberculosis and Malaria (GFATM) and the World Bank's Malaria Booster programme, most sub-Saharan African countries have now switched to the highly efficacious artemisinin-combination therapy (ACT). International initiatives such as Medicines for Malaria Venture (MMV) are increasingly speeding up the development of new anti-malarials. However, the public health impact of such drugs relies to a large extent on patient's ability to access them and little progress will be made unless broader access issues are tackled. Despite large increases in the number of anti-malarial drugs supplied internationally, surveys conducted between 2007 and 2008 in 11 African countries found that only 15% of fever cases were treated with ACT [1].

Several strategies have been proposed and tested to improve access to malaria treatment by targeting providers or users. Smith *et al *[3] recently conducted a systematic review of the effectiveness of various such interventions in improving prompt and effective treatment of malaria. Interventions reviewed included optimizing case-management and services in health facilities [4-8] or improving dispensing practices of drug shop attendants and private practitioners [9-14] as well as community based approaches [15-17]. Two general approaches to improving user malaria treatment practices have been pursued: 1) health education campaigns; [18,19] and 2) interventions that specifically provide information on how to take anti-malarials [20], including pre-packaging and pictorial and verbal instructions [21-23]. The main finding from the review is that most interventions so far have been conducted on a rather small scale and that few have been appropriately evaluated. As a consequence, despite the wealth of research conducted on the topic, little is known about interventions that can promote sustained change.

The ACCESS programme in the Kilombero and Ulanga Districts in south-eastern Tanzania aims to improve understanding of and access to prompt and effective malaria treatment through an integrated approach targeting both users and providers [24]. The programme's activities are based on a conceptual framework, which defines access as the degree of fit between the needs and means of patients (users) and the existing services (providers) along the five dimensions of availability, accessibility, affordability, adequacy and acceptability [25]. Interventions are carried out at three levels: 1) the community; 2) the formal health sector; and 3) the private retail sector for drugs. A comprehensive monitoring and evaluation plan accompanied each of these interventions. The implementation of the programme started in 2004 and the second phase will be completed in 2011

Between 2004 and 2007 the ACCESS programme's main intervention at community level was a social marketing campaign for improved recognition of the disease and more effective care-seeking. It followed on the work by the KINET project which used a social marketing approach to promote the use of insecticide treated nets in the same area [26]. Various communication channels were used and material developed to disseminate information on malaria transmission, symptoms and prevention as well as to stress the importance of prompt and effective treatment. Road shows were the main activity and included role-plays, public lectures and quizzes. In addition, promotional materials (e.g. stickers, leaflets, t-shirts) were distributed, and billboards and posters displayed in public places. The programme also organized special campaigns targeted at pregnant women and mothers of young children in mother and child health (MCH) clinics. Social marketing campaigns were conducted in 96% (78/81) of the villages in the Kilombero District and 95% (62/65) of the villages in the Ulanga District. More detailed information on the ACCESS social marketing campaigns can be found elsewhere [24].

The ACCESS programme also intervened in the public health sector to improve quality of care. Key activities included strengthening of routine supervision and refresher training for health facility staff based on integrated management of childhood illness (IMCI) Algorithms [24]. In 2004 and 2005, 91% (94/103) of all health workers in the Ulanga District and 93% (39/42) of clinical officers in the Kilombero District attended a refresher training. In addition, the study period saw the change of the first line treatment for malaria. In 2006 the Government of Tanzania switched from sulphadoxine pyrimethamine (SP) to artemether-lumefantrine (ALu). Actual introduction of ALu in health facilities was delayed until 2007, with resulting stock-outs in the transition period [27].

In parallel, the accredited drug dispensing outlets (ADDOs) programme was rolled out in the study area from 2006 onwards to improve access to treatment and quality of care in the private drug retail sector [28]. ACCESS undertook the local evaluation and monitoring of the programme. The private retail sector plays a very important role in the delivery of anti-malarial treatment in most African countries, as retailers tend to be more accessible and flexible, especially with regards to opening hours and charges [29,30]. The aim of the ADDO programme is to improve access to basic medicines by upgrading all existing drug shops to well regulated and properly operated outlets manned by specifically trained personnel [31]. The intervention involved a combination of private drug shop dispenser training, incentives, accreditation and regulation. The ADDO programme greatly improved the availability and accessibility of drug shops and, most importantly the quality of advice and dispensing [27] (Dillip et al., unpublished data). ALu was made available to the programme at a highly subsidised price towards the end of 2007, but this did not result in widespread availability of the drug, allegedly because of low profit margins on the drug and long distances to the wholesalers. Between 2006 and 2008, 55 ADDOs were opened in the Ulanga District and 135 in the Kilombero District (equivalent to approximately three shops per 10,000 people in both districts).

The primary aim of this study was to evaluate changes in understanding and treatment-seeking for malaria in the Kilombero and Ulanga Districts during the period 2004-2008 and to assess how such changes could be attributed to the three interventions evaluated by the ACCESS programme. The results presented here are complemented by a study, which focused on changes in availability, accessibility and affordability of treatment over the same period (i.e. the provider perspective) [27]. The data also provided a unique opportunity to apply a recent analytical framework on access to treatment [25] in a real-life situation and to assess the determinants of access to prompt and effective treatment.

## Methods

### Study setting

**Figure 1:**
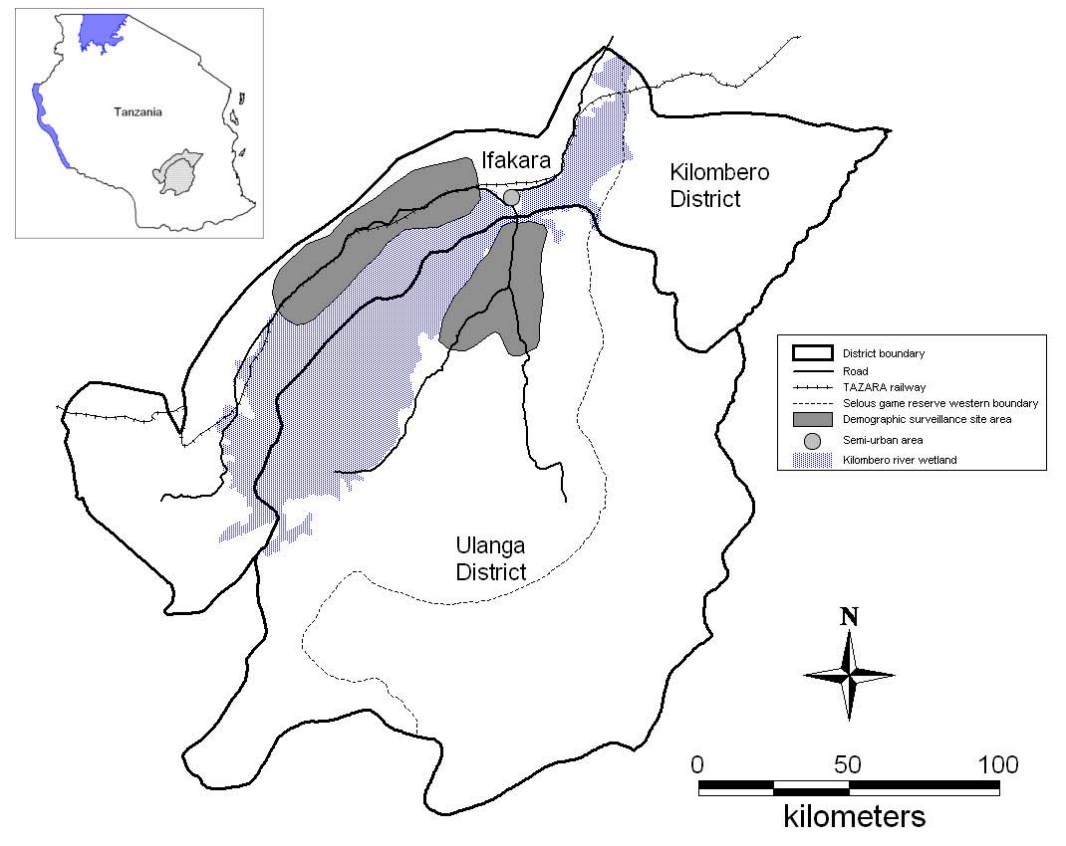
**Map of Kilombero and Ulanga Districts showing Ifakara Town and the Demographic Surveillance System (DSS)**.

The study was carried out in the rural areas of the Kilombero and Ulanga Demographic Surveillance System (DSS) and in the semi-urban setting of Ifakara town between 2004 and 2008 (Figure 1). In the rural DSS area every household is visited every four months to collect a set of basic demographic data. As a result, a comprehensive and continuously updated database of the resident population is maintained for the study area. The rural DSS covers 25 villages (13 in Kilombero and 12 in Ulanga). The population in 2004 was almost 74,000 and just over 92,000 in 2008. The population of Ifakara town was 45,700 according to the national census of 2002 [32]. A study conducted in the area between 2001 and 2003 reported an Entomological Inoculation Rate (EIR) of 349 infective bites per person per year (ib/p/y) [33], but according to recent data it has declined to 81 ib/p/y (Russell *et al*., unpublished data). EIR data for Ifakara town suggest that the transmission rate is about a log order smaller than in the surrounding rural areas [34]. The area has been described in more detail elsewhere [24,35].

#### The formal health sector

There are six health facilities in the rural Kilombero DSS area and eight health facilities in the Ulanga DSS area. The Designated District Hospital in Ifakara serves as a referral centre for the entire area and there are also two other health facilities in town. Government and faith-based facilities in Kilombero and Ulanga charge user fees. In the Ulanga District the Community Health Fund (CHF) offers a form of risk protection to its members but very few people were enrolled during the study period (around 3% of the population in 2005 - personal communication from the District Medical Officer). Children under five years of age, pregnant women and elderly people should receive services and drugs free of charge, but there is ample evidence that the exemption mechanism is not properly implemented [36,37].

#### Retail sector for drugs

By 2008 90% (49/54) of drug shops in the study area were ADDOs. Between 2004 and 2008 the number of shops per 1000 people increased from 0.24 to 0.39 and as a result the proportion of people living within 5 km from a shop increased from 71% in 2004 to 87% in 2008. The roll out of ADDOs coincided with a stark decrease in the availability of anti-malarial drugs in non-licensed general shops. Mystery shopper surveys showed that the proportion of customers with malaria symptoms who got correct advice and treatment in drug shops increased from just above 30% to nearly 80% between 2004 and 2008 (Dillip *et al*., unpublished data) after the introduction of ADDOs. Although ALu was made available in ADDOs in mid 2007 with a high level of subsidy by 2008 it was only stocked by a third of shops. SP and other older anti-malarials remained much more widely available and sold [27].

### Treatment-seeking surveys

Three cross sectional surveys were conducted in the rural DSS areas and Ifakara town in 2004, 2006 and 2008 to investigate treatment-seeking for malaria and understanding of the disease. The interviewees included children and adults who had recently suffered a fever episode (caretakers responded to questions for children under the age of 12). Data collection was carried out every other year between May and August, a time of the year which coincides with the dry season and is characterised by a lower intensity of transmission. An analysis of the baseline study in 2004 was published by Hetzel *et al *[37] and the results presented here provide a longitudinal assessment of the changes between 2004 and 2008.

#### Sampling procedure

Different sampling procedures were applied in the rural DSS area and in Ifakara Town. In the DSS area, a village-stratified random sample of households was drawn from the existing comprehensive DSS register. Only households with at least one child under the age of five years were eligible. In Ifakara such a sampling frame was not available. Therefore, the local administrative structure was used to draw a two-stage random sample of households, using ten-cell leaders (*balozi*) as first level of sampling. Given a background of decreasing fever incidence rates (Alba et al., in preparation), every year a greater number of households were sampled to ensure that approximately 150 fever cases could be followed up (Table 1). All individuals from the sampled households who reported an episode of fever within the previous 14 days were included in the study. Patients who had not recovered clinically were not included and they were instead advised to seek care from a health facility. More details on the sampling and interviewing procedure can be found in the baseline paper [37]

**Table 1:** Sample size and number of fever cases followed up in each survey round

	*DSS areas*		*Ifakara Town*	
	*Households sampled *^1^	*People interviewed*	*Households sampled* *(ten-cells × households per ten-cell*)^2^	*People interviewed*
2004	318	110	223 (40 × 6)	44
2006	561	103	410 (50 × 9)	50
2008	750	86	739 (75 × 10)	41

^1 ^Village-stratified sampling proportional to number of households per village

^2 ^Two-stage sampling of households within ten-cells. The final number of households is lower than the product of the parts as some ten-cells have less than the chosen number of households

#### Data collection tool

The tool for data collection was a locally adapted Explanatory Model Interview Catalogue (EMIC) [38] based on focus group discussions and prior research carried out in the study area [39,40]. This semi-structured questionnaire provides qualitative and quantitative data on patients' signs and symptoms associated with the fever episode (patterns of distress), as well as perceived causes and treatment-seeking. Patients were also asked to label the disease according to their own understanding. In the study area most cases were labelled as malaria, *homa *(fever) and *degedege *(fever-related disease with neurological involvement [40]).

#### Distance from households to nearest point of care

Distances from households to the nearest health facility or drug shop were calculated by combining the global positioning system (GPS) locations of households from the DSS database and the GPS positions of outlets obtained from providers surveys [27]. The households GPS values were available for 91% (100/110) households in 2004, 97% (100/103) in 2006 and 87% (73/87) in 2008.

#### Measuring socio-economic status

A relative index of socioeconomic status (SES) was calculated for the households in the DSS villages using asset ownership and household characteristics data stored in the DSS database. A principal components analysis (PCA) defined the weights of an SES index [41,42] for the households in the survey, relative to all other households in the area. Households were divided into five wealth quintiles based on their SES score. The score was available for 97% (107/110) households in 2004, 93% (96/103) in 2006 and 93% (81/87) in 2008. The variables included in the analysis and the weight given to each is shown in Table 2.

**Table 2:** Results of principal components analysis of socio-economic status (SES) variables

*Item*	*2004*		*2006*		*2008*	
	*Mean*	*weight*	*Mean *	*weight*	*Mean *	*weight*
Meals consumed per day in past 2 days	2.31	0.42				
Days per week that the following is consumed:						
Meat	0.71	0.29				
Rice	3.92	0.23				
Tea	2.37	0.42				
Main source of food:						
Market	0.52	-0.34				
Own farm	0.44	0.37				
Source of water: 1 = tap 2 = well with pump 3 = well 4 = river	2.49	-0.06				
Household owns at least 1:						
Bicycle	0.45	0.32	0.57	0.42	0.65	0.44
Radio	0.49	0.34	0.65	0.38	0.66	0.42
Animal			0.09	0.17	0.10	0.23
Mobile phone			0.07	0.28	0.28	0.42
Corrugated iron roof			0.32	0.35	0.34	0.34
Small business as source of income			0.09	0.09	0.11	0.13
Rented accommodation			0.10	-0.02	0.10	0.02
Number of mosquito nets			1.97	0.46	2.03	0.35
Number of rooms	2.09	0.17	2.18	0.45	2.23	0.38
Toilet or latrine			0.92	0.15	0.93	0.06

Number of households included	14515/14997		16762/16888		17764/18813	
Variation explained by first principal component	24%		23%		24%	

The variables collected for SES assessment in 2004 differed from the ones collected in subsequent years but this was not considered to bias analyses. Comparing SES quintile groupings in the DSS households showed similar year on year comparability across all years in the poorest and richest category. Indeed 40% (871/2157) of households categorised as poorest in 2004 were categorised as poorest in 2006 and 47% (1256/2686) households categorised as richest in 2004 were categorised as richest in 2006. Similar, albeit higher values were found comparing categories in 2006 and 2008, that is 53% (1341/2540) and 61% (1752/2882) respectively. As year on year comparability in the three middle quintiles was poor, the middle quintiles were grouped into one category which resulted in 66% (4470/6813) of household categorised as middle in 2004 also categorised as middle in 2006 and similarly 73% (5930/8104) comparing the 2006 and 2008 groupings. With these regroupings it was assumed that the difference in the type of assets collected would not introduce substantial bias.

#### Ethical clearance

All study participants provided oral informed consent prior to the interview, the National Institute for Medical Research of the United Republic of Tanzania granted ethical clearance for the study (NIMR/HQ/R.8a/Vol.IX/236, 16^th ^September 2003).

### Analyses

Various indicators were constructed and compared between 2004 and 2008. Indicators of understanding of malaria focused on perceived causes and patterns of distress. Indicators of treatment included: actions, sources and type of drugs used for the treatment of fever and links of the community effectiveness chain based on the approach developed by Hetzel *et al *[37]. The community effectiveness chain breaks down the full Roll Back Malaria (RBM) [2] indicator into its primary components [43] and includes: the proportion of fever cases 1) treated; 2) treated with a drug; 3) treated with an anti-malarial; 4) treated with a recommended anti-malarial; 5) treated with a recommended anti-malarial on the same or next day; 6) treated with a recommended anti-malarial the on same or next day and following the correct regimen (correct number of tablets, timely intake and duration), i.e. the full RBM indicator; 7) treated with a recommended anti-malarial on the same or next day, following the correct regimen and appropriately considering reported symptoms (with quinine if symptoms of severe malaria are reported). Since the change of treatment policy only effectively took place one year before the last 2008 survey, two scenarios were presented for 2008. The first scenario is strictly according to guidelines, following which only ALu qualifies as a recommended treatment, whereas the second scenario also allows for SP as a recommended treatment. Logistic regressions estimated the effect of changes over time from 2004 to 2008 with time entered in the model as a categorical variable. Estimates of change over time were reported crude as well as adjusted by SES groupings since SES score was higher in the last two surveys compared to the first survey.

The recently developed access to treatment framework [25] was applied to the data to estimate the determinants of access in the study area. The framework defines five dimensions of access, namely availability, accessibility, affordability, adequacy and acceptability. Availability refers to the existence of appropriate service. Thus, an indicator of availability was defined as the presence of an outlet (health facility or drug shop) stocking anti-malarial drugs in the village of residence of the patient. Accessibility refers to the geographical distance between the services and the homes of intended users. The main indicator of accessibility was thus defined as the distance from the patient's main residence to the nearest outlet stocking anti-malarials (health facility or drug shop). But since patients in the study area often spend a significant amount of time in farming fields far away from households and outlets [44], a secondary indicator of accessibility was defined as whether the patients were in their main residence or away in farming fields at onset of fever. Affordability refers to whether the prices of services fit the patients' income and ability to pay. The patient's SES quintile was taken as a surrogate indicator for income and how much they spent on their treatment (drug and consultation) as an indicator of their ability to pay. The data necessary to construct indicators of acceptability and adequacy were not available. The analysis only included patients from the rural DSS villages as data on SES and distances to nearest shop or health facility were not available for households in Ifakara town. Univariate logistic regressions assessed contribution of each of the access indicators on the odds of the patient receiving of prompt and effective treatment within 24 hours. A multivariate model was built (by backward elimination of variables with a log-likelihood ratio test greater than 0.2) to assess the relative contribution of each of the access indicators.

Epi Info 6 and Intercooled Stata 9 (College Station, Texas, USA) were used for random sampling procedures. Data were double entered in Microsoft FoxPro and Microsoft Access (Microsoft Corp.) and checked for coding errors and consistency. Statistical analysis was done with Intercooled Stata 9. Distance calculations were carried out with ArcMap Version 9.1 (ESRI Inc.)

## Results

The cross-sectional samples were similar over the three years of observation in terms of age, sex, residence, religion and years of formal education. Despite being marginally wealthier, households in 2008 were located further away from drug shops and health facilities (Table 3).

**Table 3:** Sample characteristics

	*2004*		*2006*		*2008*	
	*N*	n (%)*	*N*	n (%)*	*N*	n (%)*
Age group	154		153		127	
Under 5 years		81 (52.6%)		76 (49.7%)		50 (39.4%)
Over 5 years		73 (47.4%)		77 (50.3%)		77 (60.6%)
Sex	154		153		127	
Male		71 (46.1%)		63 (41.2%)		54 (42.5%)
Female		83 (53.9%)		90 (58.8%)		73 (57.4%)
Residence	154		153		153	
Ulanga DSS		61 (39.6%)		41 (26.8%)		40 (31.5%)
Kilombero DSS		49 (31.8%)		62 (40.5%)		46 (36.2%)
Ifakara		44 (28.6%)		50 (32.7%)		41 (32.3%)
Religion*	154		152		125	
Muslim		63 (40.9%)		51 (33.5%)		50 (40.0%)
Christian		91 (59.1%)		101 (66.4%)		75 (60.0%)
Years of formal education**	137		150		125	
< 7 years		56 (40.9%)		45 (30.0%)		38 (30.4%)
= 7 years		70 (51.1%)		99 (66.0%)		80 (64.0%)
> 7 year		11 (8.0%)		6 (4.0%)		7 (5.6%)
SES score ***	107		96		81	
Poorest		20 (18.7%)		6 (6.3%)		10 (12.4%)
Middle		63 (58.9%)		62 (64.6%)		52 (64.2%)
Richest		24 (22.4%)		28 (29.2%)		19 (23.5%)
SES score *** [mean (SD)]	107	0.09 (1.53)	96	0.41 (1.28)	81	0.44 (1.45)
Distance to nearest health facility (km) *** [median (IQR)]	105	1.69 (2.74)	100	1.67 (3.43)	73	2.25 (3.83)
Distance to nearest Part II or ADDO drug shop (km) *** [median (IQR)]	105	1.70 (4.13)	100	1.79 (3.30)	73	2.49 (1.94)

* unless otherwise stated

** of caretaker if patient < 12 years

*** DSS only (110 observations in 2004, 103 in 2006 and 86 in 2008)

### Understanding of malaria

**Figure 2:**
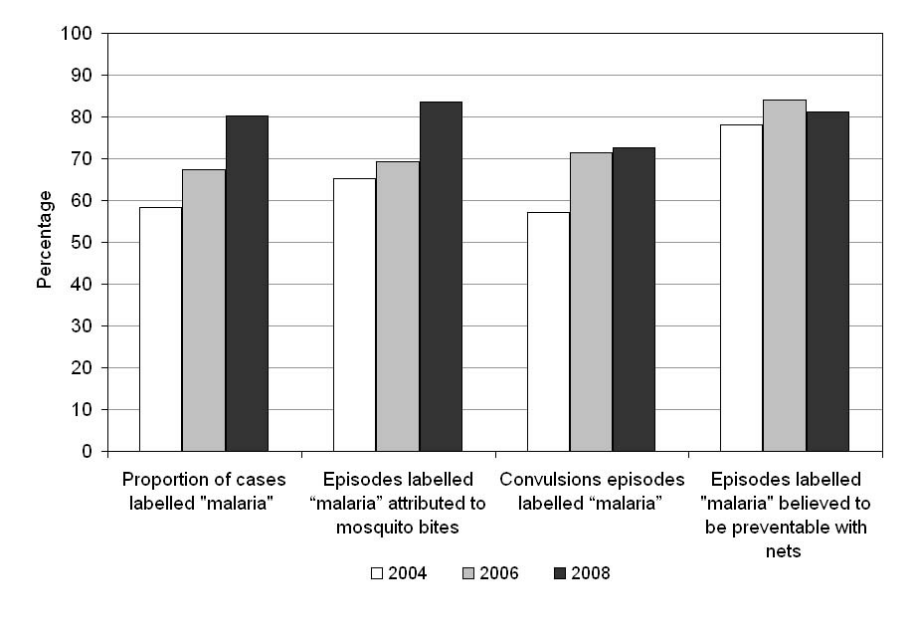
**Changes in understanding of malaria**.

The population appears to be more aware of malaria, its causes and its prevention in 2008 compared to 2004 (Figure 2). While in 2004 57% (80/137) of people labelled their fever case as malaria, the proportion rose to 80% (102/127) in 2008 (crude OR = 3.14 p < 0.001, adjusted for SES OR = 2.31 p = 0.008). The proportion of cases labelled malaria attributed to mosquito bites significantly increased from 62% (79/127) to 84% (97/116) (crude OR = 3.10 p < 0.001, adjusted for SES OR = 2.02 p = 0.056). The proportion of fever episodes with symptoms of convulsions (twitching, stiff body, delirium, white eyes, kicking limbs) labelled as malaria also consistently increased from 57% (16/28) to 73% (8/11), although not significantly so due to the small number of cases with such symptoms (OR = 2.00 p = 0.373). The proportion of fever cases believed to be preventable with the use of mosquito nets did not increase, most likely because of the already very high value of 81% at baseline.

### Treatment of fever

There was no difference in health facility attendance and treatment in children, but the proportion of older patients who sought treatment in health facilities increased significantly. Health facility attendance and treatment was already very high at baseline in children and did not change over the study period (health facility attendance at some point during the illness 148/195 i.e. 76%; health facility attendance as first treatment action 119/201 i.e. 58%; treatment with anti-malarial from a health facility 123/207 i.e. 59%). Conversely the odds of older patients attending a health facility as a first treatment option increased three-fold between 2004 and 2008 (from 20/73 i.e. 27% to 40/77 i.e. 52% OR = 2.9 p = 0.002) unadjusted and nearly five-fold adjusting for SES (OR = 4.6 p = 0.001). The odds of older patients being treated in a health facility also increased - not significantly (from 23/73 i.e. 32% to 33/77 i.e. 43% OR = 1.63 p = 0.152) unadjusted but nearly three-fold adjusting for SES (OR = 2.9 p = 0.012).

The receipt of treatment from the private retail sector increased over the study period. Overall the proportion of cases treated with an anti-malarial from a drug shop significantly increased from 31% (47/154) in 2004 to 31% (47/153) in 2006 and 43% (54/127) in 2008 (OR = 1.68, p = 0.038). Three points should be highlighted with regards to this. Firstly, this effect is confounded by SES (adjusted OR = 1.18 p = 0.632). The proportion mainly increased in the three middle wealth quintiles from 19% (12/63) to 26% (12/46) and actually decreased in the poorest quintile from 25% (5/20) to 20% (2/10) whereas it stayed stable in the richest quintile (20/74 i.e. 27%). Secondly the increase was mainly in patients over the age of five (38% in 2004 to 52% in 2008) and not in children (25% in 2005 to 28% in 2008). Thirdly the increase in the use of the private sector in patients older than five was due more to an increase in people who attended health facilities and obtained treatment from a shop (23/73 i.e. 31% in 2004 to 33/77 i.e. 43% in 2008) than in people who treated themselves directly from shops without ever visiting a health facility (23/73 i.e. 32% in 2005 to 27/77 i.e 35% in 2008). Few patients were treated from a general shop (30/434 i.e. 7%) (Table 4)

**Table 4:** Breakdown of types of anti-malarials received from each of the sources of treatment (number of cases and percentages)

	*2004*			*2006*			*2008*		
	*Health facilities*	*Drug shops*	*General shops*	*Health facilities*	*Drug shops*	*General shops*	*Health facilities*	*Drug shops*	*General shops*
Chloroquine*		1 (2.1%)							
Artemether-Lumefantrine							40 (62.5%)	7 (13.0%)	1 (16.7%)
SP	40 (58.5%)	20 (42.6%)	10 (90.9%)	41 (51.2%)	33 (70.2%)	10 (83.3%)	11 (17.2%)	35 (64.8%)	3 (42.9%)
Amodiaquine	10 (14.7%)	9 (19.2%)		16 (20.0%)	9 (19.2%)	1 (8.3%)	4 (6.3%)	4 (7.4%)	
Quinine	38 (55.9%)	26 (55.3%)	3 (27.3%)	24 (42.5%)	13 (27.7%)	3 (25%)	12 (18.8%)	11 (20.4%)	2 (28.5%)

Total	68	48	11	80	47	12	64	54	6

* 2 patients took chloroquine in 2004 but information on source of treatment was available for 1 patient

**Figure 3:**
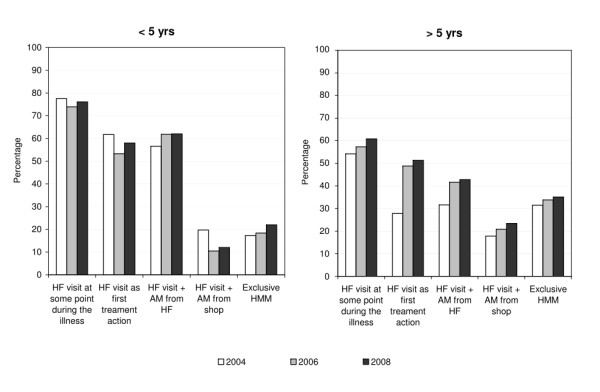
**Sources of treatment for fever and actions undertaken**. Note HF = Health facility; AM = anti-malarial; HMM = home management of malaria.

**Figure 4:**
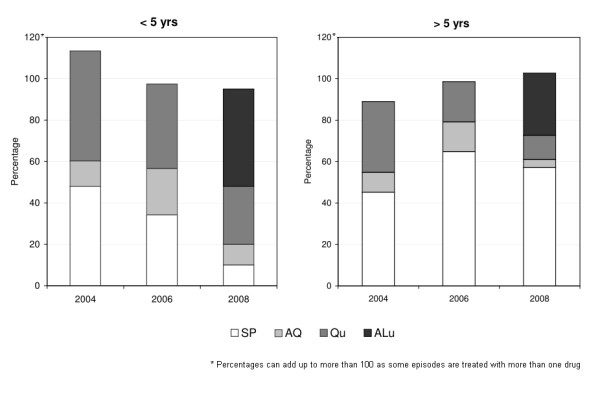
**Types of anti-malarials taken for treatment of fever**. Note SP = sulphadoxine-pyrimethamine, AQ = amodiaquine, Qu = quinine, ALu = artemether-lumefantrine.

A breakdown of the types of anti-malarials taken also shows some improvements over time although the low uptake of ALu is disappointing (Figure 3). Treatment coverage with anti-malarials is extremely high in the study area. In 2004 the use of multiple anti-malarials to treat a single fever case in children under the age of five was common but it decreased in 2006 and 2008 (Figure 4). The most commonly used drug over the study period was SP. In 2008, more than a year after the change of treatment policy, only 39% (48/124) of cases were treated with ALu. The low uptake of ALu is partly explained by the fact that even in health facilities not all cases received ALu (only 63%) and partly because most of the people who were treated in shops either received SP (65%), quinine (20%) or amodiaquine (7%) and only 13% received ALu (Table 4). Children were more likely to be treated with the new drug than older patients but not significantly so (23/50 i.e. 46% of children and vs. 23/77 i.e. 30% older patients OR = 1.28 p = 0.439). Interestingly, patients in the middle quintiles were the least likely to be treated with ALu (poorest to middle quintiles OR = 3.56 p = 0.029, richest to middle quintiles OR = 3.15 p = 0.004)

### Community effectiveness of malaria treatment

**Figure 5:**
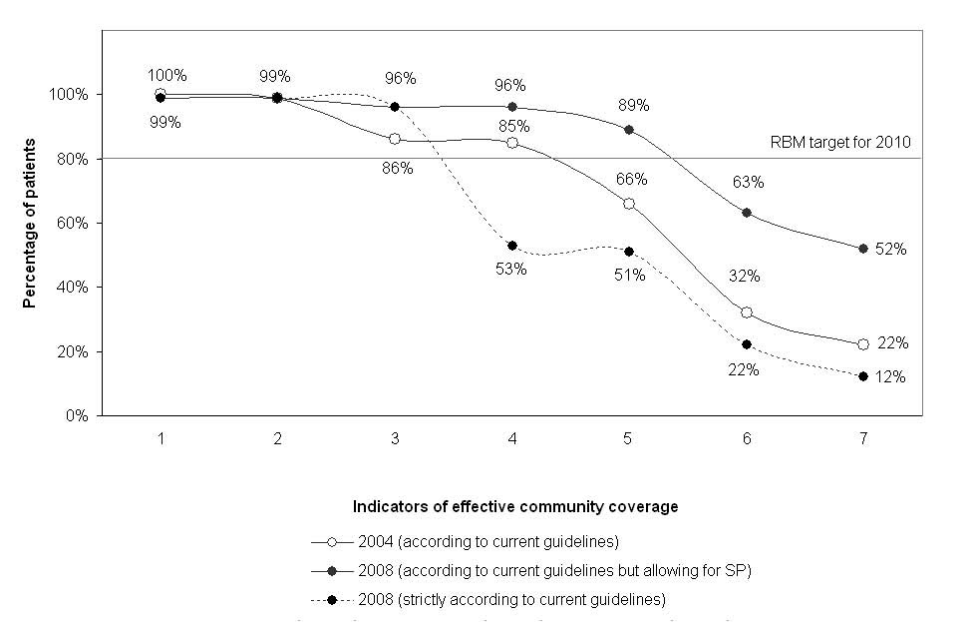
**Estimated effective coverage of fever treatment based on patients' or caretakers' accounts**. Note: Percentages are the proportion of fever cases 1) treated; 2) treated with a drug; 3) treated with an anti-malarial; 4) treated with a recommended anti-malarial; 5) treated with a recommended anti-malarial on the same or next day; 6) treated with a recommended anti-malarial on the same or next day and following the correct regimen (correct number of tablets, timely intake and duration), i.e. the full RBM indicator; 7) treated with a recommended anti-malarial on the same or next day, following the correct regimen and appropriately considering reported symptoms (quinine if symptoms of severe malaria are reported).

The comparison of the community effectiveness of malaria treatment in 2004 and 2008 shows a clear improvement over time (Figure 5). Despite a sharp fall in the proportion of people taking a recommended treatment for malaria after the switch to ALu, there were appreciable improvements in terms of timeliness of treatment. Comparing the number of people who took an appropriate anti-malarial (indicator 4) and the number who took it within 24 hours (indicator 5) shows that whereas in 2004 80% (101/127) of were treated promptly, in 2008 this figure rose to 97% (65/67) of patients who took ALu or quinine (crude OR = 8.36 p = 0.005, adjusted for SES OR = 6.39 p = 0.018), and 93% (113/122) of patients who took a ALu, quinine or SP (crude OR = 3.23 p = 0.004, adjusted for SES OR = 4.23 p = 0.015).

The self-reported adherence to the recommended drug regimen. only marginally improved. Adherence to SP treatment regimens improved over time (55/72 i.e. 76% in 2004 vs. 41/49 i.e. 84% in 2008). However, just over two thirds of patients completed their courses of ALu (22/32 i.e. 69%). Quinine treatments were always under-dosed because the course was not taken for the full seven days Overall, comparing the number of people who took an appropriate anti-malarial within 24 hours (indicator 5) and those who took it adhering full to its regimen (indicator 6) shows that in 2004 32% (32/101) of timely treatments were taken following regimen and in 2008, although this figure rose to 51% (58/113) of patients who took ALu, quinine or SP, only 31% (20/65) of patients who took quinine or SP fully adhered to their regimen (strictly according to guidelines: crude OR = 1.12 p = 0.809, adjusted for SES OR = 0.94 p = 0.910; allowing for SP as appropriate treatment in 2008: crude OR = 1.63 p = 0.212, adjusted for SES OR = 1.40 p = 0.475).

There were some difference between adults and children. Generally coverage was higher in children under the age of five compared to the rest of the population. The proportion of cases treated with a recommended anti-malarial (including SP) within 24hrs increased from 73% (59/81) in 2004 to 88% (44/50) in 2008 in children under the age of five (OR = 1.30, p = 0.030) and from 57% (42/73) in 2004 to 90% (69/77) in patients over the age of five (OR = 1.56 p < 0.001). The proportion of cases treated with either ALu or quinine within 24hrs in 2008 was 72% (36/50) in children under the age of five and 38% (29/77) in all other patients. However the proportion of cases treated promptly and effectively and following the recommended regimen tended to be slightly lower in children under five than in adults. Allowing for SP the proportion in children under five increased from 37% (16/43) to 56% (19/34) and in adults from 28% (16/58) to 67% (39/58) between 2004 and 2008. Strictly according to guidelines, i.e. excluding SP and conforming to the full RBM indicator for prompt and effective treatment of malaria, the figure in 2008 is 39% (13/33) for children and 12% (7/58) in adults.

### Determinants of access

An analysis of the contribution of each of the access dimensions suggests that the **availability **of anti-malarials, i.e. the presence of a drug outlet (health facility or drug shop) in patients' village of residence, is the main determinant of whether people get prompt and effective treatment for malaria (Table 5). There was no significant difference in promptness of treatment between patients who had been treated in health facilities and those treated in drug shop (OR = 1.28 p = 0.613), but patients treated in drug shops were less likely to be treated with the appropriate drugs (OR = 0.14 p = 0.001). Patients living in villages with either a drug shop or a health facility were four times as likely to get prompt and effective malaria treatment than people from villages without outlets (OR = 4.10 p < 0.001 adjusting for differences in affordability and accessibility). The presence of outlets influenced promptness (OR = 5.83 p < 0.001) and appropriateness (OR = 4.75 p < 0.001) of treatment to a similar extent.

**Table 5:** Determinants of receiving prompt and effective anti-malarial treatment according to current guidelines from either a health facility or a drug shop in the rural DSS villages between 2004 and 2008

		*Univariate model*			*Multivariate model (n=264)*	
		*n*	*OR* (95% CI) *	*p*	*OR* (95% CI) *	*p*
Availability	Presence of outlet in the village of residence **	297	**3.32** **(1.96 to 5.63)**	**< 0.001**	**4.10** **(2.17 to 7.73)**	**< 0.001**

Affordability	SES (baseline: middle quintiles)	282		0.951		
	Poorest		0.94 (0.44 to 2.03)	0.893		
	Richest		1.07 (0.59 to 1.95)	0.803		
	Cost of treatment *** (TSh1000)	297	**1.73** **(1.26 to 2.43)**	**0.002**	**1.74** **(1.16 to 2.60)**	**0.008**

Accessibility	Distance household to nearest outlet ** (1 km)	276	**0.84** **(0.73 to 0.95)**	**0.006**	0.88 (0.75 to 1.01)	0.082
	Location at onset of fever (home vs. farming site)	295	**1.83** **(1.03 to 3.27)**	**0.039**	**2.08** **(1.07 to 4.09)**	**0.032**

* Adjusted for the effect of year of study

** Presence/distance to health facility for those treated in a health facility and presence/distance to drug shop for those treated in a drug shop

*** Drug + consultation

**Affordability **contributed to a lesser extent to receipt of treatment. Both the univariate and multivariate model showed no difference in receipt of prompt and effective treatment across socio-economic groups. However, people who paid more for their treatment were more likely receive it promptly and effectively (OR = 1.74 p = 0.008). This implies that even poorer people manage to find the resources to afford treatment. The cost of treatment influenced the appropriateness of treatment (OR = 3.34 p < 0.001) more than the timeliness (OR = 1.67 p = 0.017). It is worth recalling here that a somewhat un-expected pattern of ALu uptake was observed in 2008 whereby the three middle quintiles were less likely to be treated with ALu (cf. results in "Treatment of fever").

**Accessibility **is a determinant of access to treatment, but only if people are in far away farms at onset of their disease. People were twice as likely to be treated promptly and effectively if they were residing in their main homestead rather than in the farming fields at the onset of their fever (OR = 2.08 p = 0.032 in the multivariate model). The location at onset of disease mainly influenced the receipt of a recommended anti-malarial (OR = 2.48 p = 0.004) rather than the timeliness of treatment (OR = 1.63 p = 0.133). However, if people were in their main homestead at onset of disease, the distance to the nearest outlet did not have a major impact on treatment provided the outlet was present in their village (OR = 0.88 p = 0.082).

## Discussion

The results presented here show improvements in understanding and treatment-seeking for malaria in two rural districts in Tanzania after the implementation of the ACCESS programme. Specific improvements include a better understanding of the causes of malaria, an increase in health facility attendance and treatment in patients older than five years, and more timely use of anti-malarials. Unfortunately, the change of malaria treatment policy from SP to ALu during the same period led to a lower availability of the first line drug in the private retail sector. As a consequence the proportion of patients taking a recommended malaria treatment dropped significantly in 2008.

Given the before-after nature of this study and the absence of a control group it is difficult to attribute specific improvements to ACCESS interventions. However, plausible explanations can be given on the basis of accepted frameworks [45,46]. With this type of design improvements are attributed to the programme if improvements are found in every step of the causal pathway between intervention and outcomes and all other explanations can be formally discarded. In this study the main outcome of interest is prompt and effective treatment and steps in the causal pathway include: 1) changes in understanding of malaria; 2) changes in actions for the treatment of malaria and; 3) changes in access outputs, i.e. the accessibility, availability and affordability of treatment, which were reported in a separate study [27].

It seems reasonable to credit the ACCESS social marketing campaigns for part of the observed changes in the understanding of malaria and the increased use of health facilities as a first treatment action. Indeed, no other efforts of this magnitude took place concurrently in the area although there were some national campaigns on both TV and radio, which stressed the importance of prompt treatment for malaria. With regards to the lack of increase in health facility attendance in children, it is important to bear in mind that health facility attendance in this age group was already very high for a rural African setting with over 75% of children visiting a facility at some point during their illness. The high level of action already undertaken by mothers of small children at the start of ACCESS is undoubtedly the result of a long-standing effort in the area to improve comprehensively malaria control parameters [26,47]. It is especially worthy of note that increases in health facility attendance were higher once adjusted for SES status. This would suggest the ACCESS social marketing campaigns were able to target even the less well off, which is far from always being the case [48]. This result is consistent with data from the evaluation of the Tanzania National Voucher Scheme, which found that road shows are able to disseminate messages more equitably than any other means of communication such as radios and billboards (Hadji Mponda, unpublished data).

The more timely use of anti-malarials can be attributed to the combination of the ACCESS social marketing campaigns and the ADDO intervention. On one hand the ACCESS social marketing campaigns stressed the importance of prompt and effective treatment for malaria. On the other hand following the ADDO intervention treatment became more available and accessible [27]. The good availability of ALu in health facilities in 2008, combined with the increase in health facility visits as a first treatment action is likely to also have contributed to the improved timeliness of treatment. It is worth highlighting here two positive outcomes with regards to the role of ADDOs and health facilities, which emerged from this evaluation. Firstly self-treatment at home did not increase significantly in neither children nor older patients suggesting that ADDOs did not undermine the role of the formal health sector, but rather complemented it. Secondly, after the introduction of ADDOs, the proportion of children who were taken to a health facility and received an anti-malarial from a shop did not change (10-12%), which refutes claims that health facility staff refer patients to outlets in which they have a financial stake. It is important the ADDOs continue to be adequately supervised for these gains to be sustained.

The results presented here are largely compatible with findings from the Smith *et al*. review [3], which concluded that interventions involving private sector providers generally show a good impact. Similar to the Kilifi Shopkeeper programme in Kenya [9,12] and other studies targeting the private retail sector [10,13,14] (but contrary to results by Winch *et al*. in Mali [16]), the ADDO programme has achieved notable gains in terms of the quality of advice given by shopkeepers. Ignoring the treatment policy change, the proportion of patients treated promptly and effectively according to the recommended regimen in both the private retail sector and the public health sector increased from 32% to 63% in the present study. This increase is comparable to the outcomes in Kilifi (from 2% to 28% treated promptly and effectively with the right dosage and duration) or in Mali (from 2% to 42% treated effectively with the right dosage and duration), which only targeted the private retail sector. However, this study has shown for the first time that improvements can be sustained even at much higher levels by focusing on both on the private and the public sector and that the 80% levels targeted by the RBM Partnership are within reach [2].

The results presented here also confirm the reviewers' conclusion that improving user practice is more challenging. Noticeable gains were made in terms of people's actions for the treatment of fever (health facility attendance for patients older than five years, timeliness of treatment) but there was no clear improvement in the proportion of cases following the recommended regimen of anti-malarials. This appears to be the result of poor patient adherence (mystery shopper surveys showed that the proportion of customers with malaria symptoms who got correct advice and treatment in drug shops increased from just above 30% to nearly 80% between 2004 and 2008, Dillip *et al*, unpublished data). It is likely to be due to the introduction of a new treatment (ALu) with a more complicated regime than the previously recommended SP combined with the absence of messages targeted at users specifically focusing on the importance of adhering to treatment regimens (although ALu was pre-packaged with clear instructions and pictograms). Future social marketing campaigns in the area should put more emphasis on this component.

The community effectiveness chains show that treatment coverage with anti-malarials is very high in the study area, but the change of treatment policy led to a significant drop in the proportion of patients taking a recommended malaria treatment. Despite high levels of ALu stock in health facilities, the proportion of patients treated with ALu remained low partly because the drug was not widely available in drug shops. However, the decrease in the proportion of patients treated strictly according to guidelines should be seen in the light of the fact that already in 2003 SP had a treatment efficacy of 50% [49], while upon its introduction ALu had a treatment efficacy of more than 95% [50]. The low availability of the first line drug in the private retail sector following the change of treatment policy from SP to ALu, coupled with the multiple stockouts of SP in public health facilities during the treatment transition clearly indicate that malaria treatment policy changes need to be considered in a more comprehensive way. Fortunately, the availability of ACTs in the commercial retail sector will be considered in Tanzania in 2010 in the frame of the Affordable Medicine Facility for malaria [51].

Strategies to improve access to treatment should focus especially on the availability of points of care. The presence of a health facility or drug shop in the village of residence was the strongest predictor of prompt and correct treatment, whereas affordability (within the observed range of prices) and accessibility indicators contributed to a lesser extent. These results differ from those presented in a paper, which focused specifically on the farming season, and according to which fever cases which occurred in the farming sites were as less likely to be treated promptly and effectively as those which occurred in the main homestead [44]. This discrepancy is probably due to design issues (different populations and different season). But the considerations from the analysis presented here are largely consistent with a qualitative study on livelihood and health care which revealed that patients make considerable efforts to access treatment, including walking long distances and selling important livelihood assets [52]. Hence future interventions aimed specifically at improving access to treatment should focus on extending the network of health facilities and ADDOs to underserved villages and ensuring that drugs are available.

The main limitation of this study is the selection of cases on the basis of reported fever only, since not every fever case is due to malaria. Considering every fever as a potential malaria case is consistent with the Integrated Management of Childhood Illness (IMCI) guidelines for treatment in areas of stable malaria [53]. This approach was warranted when the survey was designed in 2003 and the area experienced very high levels of transmission. This is no longer justified in the study area since: 1) recent data suggest a drastic change in the epidemiology of malaria in the study area; and 2) a recent study piloting the use of Rapid Diagnostic Tests for malaria (mRDT) in local health facilities found that only 40% of fever cases were actually due to malaria (D'Acremont *et al*. unpublished data). It is difficult to assess how much the presence RDTs at the time of our surveys would have changed our observations.

Another limitation concerns the variability observed in the samples and the inconsistency of a finding with another study conducted in the area. Despite a random sampling from the comprehensive DSS database households in 2008 were located further away from health facilities and drug shops despite an increase in the accessibility of these outlets [27]. Furthermore the distribution of patients across SES appeared to be unequal over the years, with wealthier households over-represented in the 2006 and 2008 samples. To account for differences in SES across years all ORs of significant effects were reported crude and adjusted for SES.

Finally, it is worth noting additional positive patterns of treatment emerged from this analysis which were not consistent with conclusions one might draw from the provider survey conducted in the study area. Firstly, no patient in Ifakara reported using an artemisinin monotherapy although they were available in 10-20% of shops [27]. Secondly, the severe stockouts of SP, which were reported by health facilities in the study area in 2006 did not appear to have an impact on treatment since the proportion of patients who obtained the drug did not decrease as a result. The reason for this apparent paradox is that availability data from health facilities is based on end-of-month balance in the store-rooms. Hence, stocks may be delivered at the beginning of the month and dispensed in their entirety by the end of the month or drugs may not be in the store-room but still present in the dispensing room.

## Conclusions

An integrated approach aimed at improving understanding and treatment of malaria has led to tangible improvements in terms of people's perception and actions for the treatment of malaria. The positive results testify that even in a poor and remote African setting, the Abuja targets for access to treatment can be achieved. A higher impact on prompt and effective treatment according to treatment guidelines was hindered by a change of treatment policy, which led to low availability of the first-line drug (ALu) in the private retail sector. This shows clearly that ensuring consistent stocks of ALu in the private retail sector is crucial to improve prompt and effective treatment for malaria. Future interventions aimed at improving access to treatment by targeting users should have a focus on adherences to treatment regimens. Interventions targeting providers should aim at extending the network of health facilities and ADDOs to underserved villages.

## Competing interests

AS is employed by the Novartis Foundation for Sustainable Development (NFSD) which funded the ACCESS programme. The Foundation works independently from the company's business and supports not-for-profit health programmes in developing countries.

## Authors' contributions

SA participated in the supervision of the data collection in 2008, analysed the data, drafted and finalised the manuscript. AD participated in the supervision and training for the data collection in 2006 and 2008 as well as contributing to the manuscript. MWH participated in the design of the survey, coordinated the data collection in 2004 and 2006 and contributed to the manuscript. CM and AM designed and conducted the social marketing campaigns. MA was responsible for the DSS, performed the sampling for the DSS surveys and supervised the data collection. IM participated in the data collection and contributed to the discussion on the manuscript. BO and AS participated in the design of the surveys and contributed to the discussion on the manuscript. HM and FK provided overall coordination and contributed to the discussion on the manuscript. CL contributed to the study design, the data analysis and the manuscript. The final manuscript was approved by all authors.
